# Whole-Genome Analysis of Recurrent *Staphylococcus aureus* t571/ST398 Infection in Farmer, Iowa, USA

**DOI:** 10.3201/eid2401.161184

**Published:** 2018-01

**Authors:** Shylo E. Wardyn, Marc Stegger, Lance B. Price, Tara C. Smith

**Affiliations:** University of Iowa, Iowa City, Iowa, USA (S.E. Wardyn);; Statens Serum Institut, Copenhagen, Denmark (M. Stegger);; Translational Genomics Research Institute, Flagstaff, Arizona, USA (L.B. Price);; George Washington University, Washington, District of Columbia, USA (L.B. Price);; Kent State University, Kent, Ohio, USA (T.C. Smith)

**Keywords:** methicillin-resistant Staphylococcus aureus, MRSA, methicillin-sensitive S. aureus, MSSA, livestock, swine, antimicrobial resistance, farming, farmer, zoonosis, whole-genome analysis, sequence types, bacteria, Iowa, United States

## Abstract

*Staphylococcus aureus* strain sequence type (ST) 398 has emerged during the last decade, largely among persons who have contact with swine or other livestock. Although colonization with ST398 is common in livestock workers, infections are not frequently documented. We report recurrent ST398-IIa infection in an Iowa farmer in contact with swine and cattle.

Livestock, especially swine, are a reservoir for *Staphylococcus aureus* sequence type 398 (ST398) (*1*). Carriage of this strain is primarily reported in persons with occupational exposure to livestock; however, less is known about the frequency and severity of ST398 infections, particularly in the United States, where surveillance for this organism is limited (*2*). We report colonization and recurrent infection with methicillin-sensitive *S. aureus* (MSSA), belonging to livestock clade ST398, in a farmer in Iowa, USA.

A participant in a longitudinal study of rural Iowans (*2*) provided swab samples of his current skin infection. Swabs were cultured, and *S. aureus* isolates were subjected to molecular analyses as previously described (*3*,*4*). Institutional review board approval was obtained from the University of Iowa.

The 61-year-old man enrolling in the Iowa study in July 2011 reported a skin infection on his foot. He had visited a doctor the previous day and received ciprofloxacin, and noted his infection was chronic. He also reported a history of heart disease and diabetes. The participant was a farmer who raised swine (900 head × 40 years) and cattle (500 head × 10 years), and owned a dog. He reported working directly with swine and cattle ≈2 hours per day and noted that he did not use personal protective equipment such as gloves or masks. His nose and throat were colonized with MSSA ST398 that was *mecA*-negative, periventricular leukomalacia–negative, and staphylococcal protein A type t571. The isolate was resistant to tetracycline, trimethoprim/sulfamethoxazole, and levofloxacin. The participant’s wife was also colonized at enrollment with MSSA in her throat, showing the same molecular characteristics and susceptibility patterns as her spouse. She reported no exposure to livestock.

The isolate obtained from the participant’s first culture showed the same molecular characteristics as isolates from his nasal and throat swabs and the isolate from his second infection culture received in November 2011 (Table). He described this second infection as cellulitis: he reported draining pus, a general ill feeling, and headaches. The infection was treated empirically with ciprofloxacin, neosporin, warm compresses, and incision and drainage. Isolates from his third incident infection culture in August 2012 showed the same molecular characteristics as previous samples, but 2 different susceptibility patterns: 1 isolate was the same as previous, while another showed additional phenotypic resistance to oxacillin (MIC  4 µg/mL by broth dilution), making this a borderline-resistant *S. aureus* infection. Genome sequence analyses showed that this strain had not acquired a *mec* gene and likely had become resistant from a mutation in a penicillin-binding protein. This infection was treated with mupirocin, trimethoprim/sulfamethoxazole, ciprofloxacin, warm compresses, and drainage.   All of the farmer’s isolates had the t571 *spa* type, which is common among livestock-independent (i.e., human-associated) ST398 lineages (*4*). *S. aureus* t571 strains have been isolated from an Iowa childcare worker (*5*), inmates from a Texas jail (*6*), and in community members in New York and New Jersey (*7*); t571 strains have been rarely isolated from Iowa livestock (*8*). However, whole-genome sequence analyses with 89 previously published clonal complex 398 genome data confirmed that the isolates from this case were from the livestock-associated ST398 lineage (CC398-IIa) (*4*).

**Table:** Details of recurrent *Staphylococcus* *aureus* t571/ST398 infection in farmer, Iowa, USA*a

Date	Antimicrobial drug resistance profile	Description of infection	Treatment
July 2011	TET, SXT, LVX	Cellulitis	Ciprofloxacin
November 2011	TET, SXT, LVX	Cellulitis, draining pus, headache, ill feeling	Ciprofloxacin, Neosporin, warm compress, incision, drainage
August 2012†	TET, SXT, LEVO (isolate 1); OXA, TET, SXT, LVX (isolate 2)	None provided	Mupirocin, SXT, ciprofloxacin, warm compress, drainage

*All isolates were t571/ST398 and were PVL negative. Neosporin is manufactured by Johnson & Johnson (New Brunswick, NJ, USA). LEVO, levofloxacin; LVX, levofloxacin resistance to isolate 2; OXA, oxacillin; SXT, trimethoprim/sulfamethoxazole; TET, tetracycline. †Two isolates from this infection were tested and had different antimicrobial drug resistance profiles as noted.

Longitudinal follow-up complemented with whole-genome sequence analyses showed that the participant repeatedly experienced infections by the same strain over the course of 13 months. The isolates from this patient and his wife formed a distinct clade within the ST398 global phylogeny (Technical Appendix Figure 1). In-depth genomic analyses revealed a novel ≈265,000 bp recombinant region originating from clonal complex 9, and unique to this lineage.

It is unclear whether the farmer’s recurrences arose from treatment failure, repeated acquisitions from exposure to livestock, or the result of long-term colonization and evolution. In contrast, the farmer’s wife, who was colonized by the same strain, reported no direct contact with livestock and may have become colonized by human-to-human transmission from her husband. Comparison with other strains collected as part of this (*2*) and other studies (*9*) identified closely related isolates from 3 other participants and 1 pig (Technical Appendix Figure 1) distributed throughout the state of Iowa and into western Illinois (Technical Appendix Figure 2).

This dataset does not specify whether the evolution of the borderline-resistant *S. aureus* phenotype took place in the patient or the livestock reservoir. The farmer was prescribed ciprofloxacin multiple times; this could have contributed to emergence of borderline oxacillin resistance (*10*). The farmer’s first phenotypically methicillin-resistant strain was not detected until >1 year after enrollment into this study. This case demonstrates the ability of livestock-associated *S. aureus* ST398 to cause repeated skin and soft tissue infections in a manner similar to community-associated strains, and the necessity to screen for MSSA and methicillin-resistant *Staphylococcus* *aureus* when studying the spread of these organisms.

**Technical Appendix.** Maximum-likelihood phylogenetic analysis of sequence type 398 strains based on core-genome single-nucleotide polymorphisms and locations of study participants and swine in Iowa and western Illinois, USA
