# Supplementary material for: Whole-Genome Analysis of Recurrent Staphylococcus aureus t571/ST398 Infection in Farmer, Iowa, USA
Source: Emerg Infect Dis. 2018 Jan;24(1):153–4. doi: 10.3201/eid2401.161184 (PMC5749462; doi:10.3201/eid2401.161184)
Supplement: Supplementary file 1 — Technical Appendix. Maximum-likelihood phylogenetic analysis of sequence type 398 strains based on core-genome single-nucleotide polymorphisms and locations of study participants and swine in Iowa and western Illinois, USA [file 16-1184-Techapp-s1.pdf]

Article DOI: https://doi.org/10.3201/eid2401.161184

Whole-Genome Analysis of Recurrent *Staphylococcus aureus* t571/ST398 Infection in Farmer, Iowa, USA

Technical Appendix

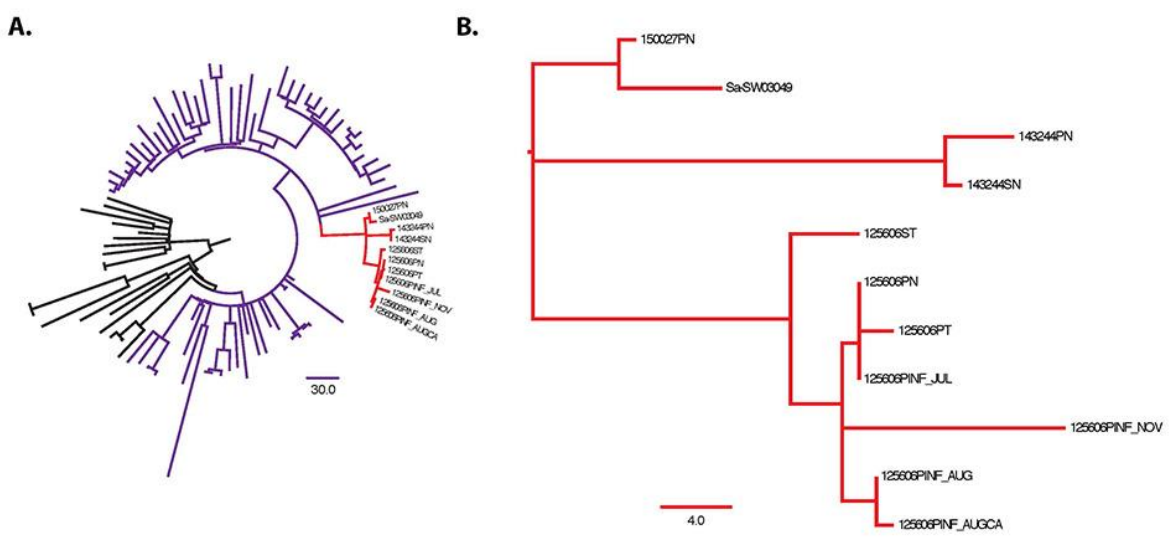

Technical Appendix Figure 1. Phylogenetic analysis of ST398 strains of *Staphylococcus aureus*, Iowa, USA. Maximum-likelihood phylogenetic trees were generated based on core-genome single- nucleotide polymorphisms. A) Isolates from the current study (red) are shown in context with a well-defined collection of ST398 from humans and livestock (*4*). All strains from the current study are clustered within the livestock clade (purple) and separate from the livestock-independent human isolates (black). B) High-resolution phylogenetic analysis shows that epidemiologically linked isolates clustered most closely to one another. Participant (PT), spouse (SP), pig (SW), nose (N), throat (T), infection (Inf), November (Nov), July (Jul), August (Aug). Scale bars indicate nucleotide substitutions per site. ST, sequence type.

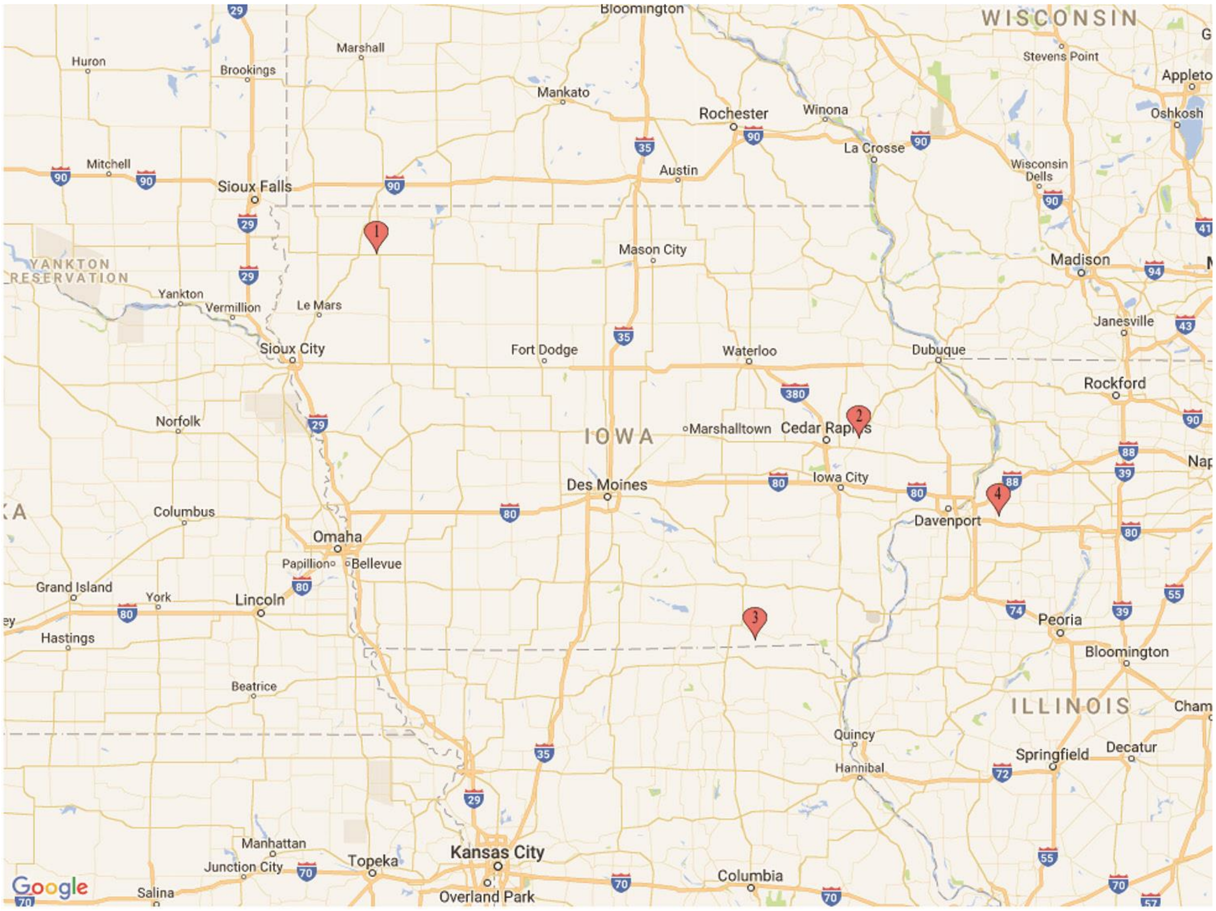

Techncial Appendix Figure 2. Geographic locations of participants (pins 1–3) and swine (pin 4), Iowa, USA.
